# Long intergenic non-coding RNA expression signature in human breast cancer

**DOI:** 10.1038/srep37821

**Published:** 2016-11-29

**Authors:** Yanfeng Zhang, Erin K. Wagner, Xingyi Guo, Isaac May, Qiuyin Cai, Wei Zheng, Chunyan He, Jirong Long

**Affiliations:** 1 Division of Epidemiology, Department of Medicine, Vanderbilt University Medical Center, Nashville, TN 37203, USA; 2 Department of Epidemiology, Richard M. Fairbanks School of Public Health, Indiana University, Indianapolis, IN 46202, USA; 3 Bowdoin College, Brunswick, ME, 04011, USA; 4 Indiana University Melvin and Bren Simon Cancer Center, Indianapolis, IN 46202, USA

## Abstract

Breast cancer is a complex disease, characterized by gene deregulation. There is less systematic investigation of the capacity of long intergenic non-coding RNAs (lincRNAs) as biomarkers associated with breast cancer pathogenesis or several clinicopathological variables including receptor status and patient survival. We designed a two-stage study, including 1,000 breast tumor RNA-seq data from The Cancer Genome Atlas (TCGA) as the discovery stage, and RNA-seq data of matched tumor and adjacent normal tissue from 50 breast cancer patients as well as 23 normal breast tissue from healthy women as the replication stage. We identified 83 lincRNAs showing the significant expression changes in breast tumors with a false discovery rate (FDR) < 1% in the discovery dataset. Thirty-seven out of the 83 were validated in the replication dataset. Integrative genomic analyses suggested that the aberrant expression of these 37 lincRNAs was probably related with the expression alteration of several transcription factors (TFs). We observed a differential co-expression pattern between lincRNAs and their neighboring genes. We found that the expression levels of one lincRNA (RP5-1198O20 with Ensembl ID ENSG00000230615) were associated with breast cancer survival with *P* < 0.05. Our study identifies a set of aberrantly expressed lincRNAs in breast cancer.

The advances in high-throughput sequencing technologies provide a systematic landscape in the complexity of transcriptome, especially the discovery of a wealth of long non-coding transcripts^1^,^2^. This phenomenon is referred to as pervasive transcription^3^, albeit some transcripts are probably the byproduct of the transcription noise^4^. Characterized as transcription from the intergenic regions, lincRNAs are specific types of long non-coding RNAs (lncRNAs), ranging in length from 200 nt to >100 kb^5^. Biochemically, many lincRNAs harbor mRNA-like features, such as the poly-adenosine (polyA+) tail and splicing^6^, enabling them to be detectable by polyA+ RNA-sequencing (RNA-seq) method. Functionally, there is mounting evidence that lincRNAs may act as modular scaffolds to recruit other active or repressive regulators^7^, resulting in widespread roles in imprinting control^8^, chromosome remodeling^9^, pluripotency and differentiation^10^, immune responses^11^ and cancer progression^12^. Many lincRNAs are expressed in high tissue or cell specificity^10^, serving lincRNA molecules as potential disease biomarkers.

Recently, the Cancer Genome Atlas (TCGA) has revealed that the aberrant expression of hundreds of protein-coding genes and tens of microRNAs are associated with breast cancer development, progression, metastasis^13^,^14^,^15^,^16^ and subtypes^17^,^18^. One large-scale study also reported hundreds of lincRNAs showing expression changes in breast cancer^19^, but this study has a lack of replication and comprehensive analyses. To further use lincRNAs as robust biomarkers, we performed an integrative analysis on RNA-seq data from approximately 1,000 breast tumors from the TCGA and an independent RNA-seq dataset of 50 pairs of matched tumors and adjacent normal tissues from breast cancer patients as well as 23 normal breast tissues from healthy women.

## Results

### Data summary

In the discovery stage, RNA-seq data for 85 pairs of breast tumors and adjacent normal tissues were collected, along with 830 primary breast tumors available from TCGA. In the replication stage, RNA-seq data from paired tumor and adjacent normal tissues from 50 breast cancer patients and normal breast tissue from 23 healthy women were included. Overall, the study subjects had similar clinical characteristics between the two stages (Table 1).

Also included in the present study were Genome-wide protein-DNA interaction data using chromatin immunoprecipitation followed by sequencing (ChIP-seq) for 26 transcription factors (TFs) in MCF-7 cell lines from the ENCODE project, and two independent ChIP-seq datasets for transcription factor ERα each with replicated experiments in the MCF-7 cells^20^,^21^, as well as RNA-seq data across 14 breast cancer cell lines (Table S1).

### Global changes of the lincRNA species in breast cancer

**Figure 1:**
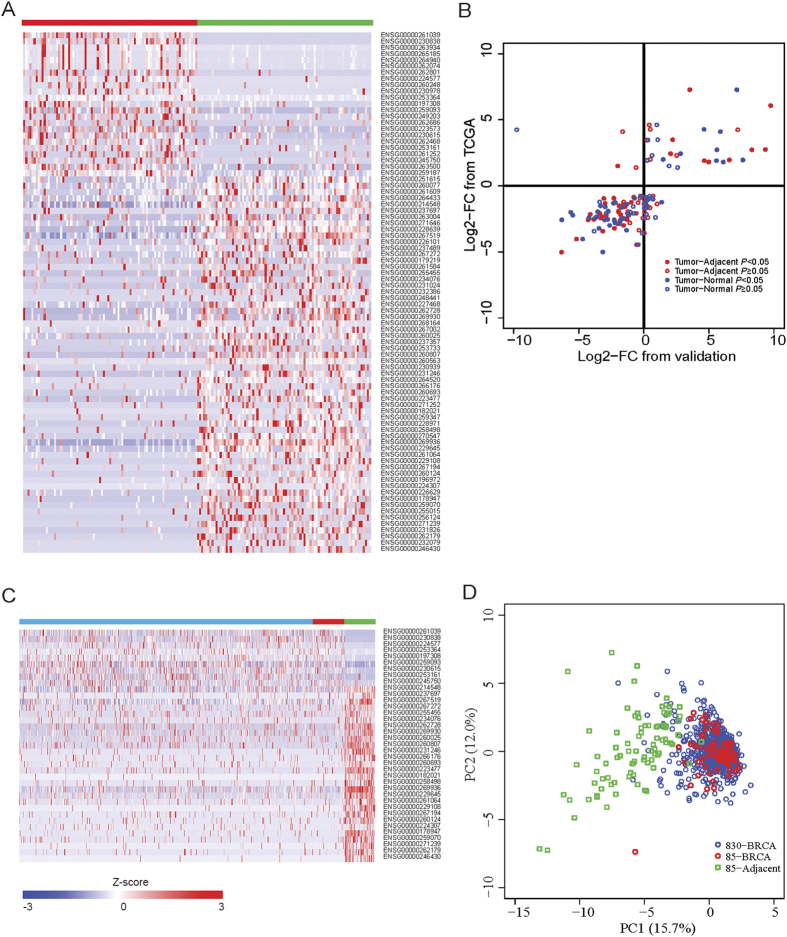
Expression differences of lincRNAs between human breast cancer and normal tissues. (**A**) Heatmap of 83 differentially expressed lincRNAs in 85 pair-matched breast cancer samples. Red and green bars denote 85 breast cancer specimens and adjacent normal tissues from TCGA, respectively. Colors ranging from blue to red represent the relative expression levels (Z-score) of lincRNAs. (**B**) Dot plot of the Log2-transformed FC for 83 lincRNAs between 50 paired breast tumor and adjacent normal tissues (Tumor−Adjacent), and between 50 breast tumors and 23 normal tissues from healthy women (Tumor−Normal) as validation (X-axis) versus that from TCGA breast cancer patients (Y-axis). (**C**) Heatmap of 37 differentially expressed and replicated lincRNAs in 830 unmatched tumors (blue bar) in combined with 85 pairs of tumors (red bar) and adjacent normal tissues (green bar). Values range in color from blue to red, meaning the relative expression levels. (**D**) PCA of 85 paired breast cancer and adjacent normal samples (85 BRCA and 85 Adjacent) and 830 unpaired breast cancer samples (830 BRCA) based on 37 differentially expressed lincRNAs.

Our transcriptomic analyses focused on the lincRNA species, a specific type of lncRNAs. First, we investigated the lincRNA expression profile in 85 paired samples of primary breast tumor tissue and adjacent normal tissue from TCGA. After stringent filtering (see Methods), we totally identified 2,171 lincRNAs expressed in at least one sample. As expected, most lincRNAs expressed at low levels and sparsely expressed in <10% of all samples (Figure S1). Similar observations were also reported elsewhere^22^,^23^,^24^. For subsequent analysis, we focused on 584 lincRNAs that expressed in at least 20% of samples. Compared with the matched adjacent normal tissues, we observed that these lincRNAs showed a global decreased expression profile in breast tumors (Figure S2). The similar trend has been reported for small non-coding RNAs in multiple cancer types^25^,^26^. Specifically, among these 584 lincRNAs, 83 were significantly differential expression (DE) with a permutation-based FDR < 1% (Fig. 1A). Among them, 60 lincRNAs displayed down-regulation in breast tumor tissues.

### Validation of lincRNA expression in an independent study

To validate the DE lincRNAs identified in TCGA, we generated an independent RNA-seq dataset from 50 pairs of matched tumor and adjacent normal samples from breast cancer patients and 23 normal breast tissues from healthy women in the replication study. Among the 83 DE lincRNAs, 66 lincRNAs (80%) showed consistent expression change direction (Fig. 1B). Specifically, 37 lincRNAs (45%) were replicated with absolute log2-FC ≥ 1 and *P *< 0.05 in the 50 pairs of matched tumor and adjacent normal breast tissues in replication stage (Table 2). Over 80% of them also showed significant expression difference in the tumors compared to normal breast tissues in the validation stage (Table 2). When we relaxed the cutoff to absolute log2-FC ≥ 0.585 and *P *< 0.05 in the replication dataset (Table S2), 47 DE lincRNAs (57%) could be replicated in our independent study, which is consistent with the previous report^19^.

We further examined the expression profiling of the 37 replicated lincRNAs in the remaining 830 breast tumor tissues samples without matched normal tissues from the TCGA dataset. The expression patterns of these 37 lincRNAs in the 830 tumor samples were very similar to those in 85 normal-paired breast tumor samples, but very different from those in adjacent normal breast tissue samples in the discovery stage (Fig. 1C). Principal component analysis (PCA) based on these 37 lincRNAs also showed that all breast tumor samples are clustered together, but separated from the adjacent normal tissues (with a few exceptions) (Fig. 1D). In addition, we also checked the expression levels of these 37 lincRNAs across 14 breast cancer cell lines that are commonly used in the lab (Table S3). Most of these 37 lincRNAs were expressed in at least one common breast cancer cell line, making these cell lines as possible *in vitro* model to study lincRNAs. Altogether, these results suggested that these 37 lincRNAs showed aberrant expression in breast tumors.

### Association of LincRNAs with transcription factors

**Figure 2:**
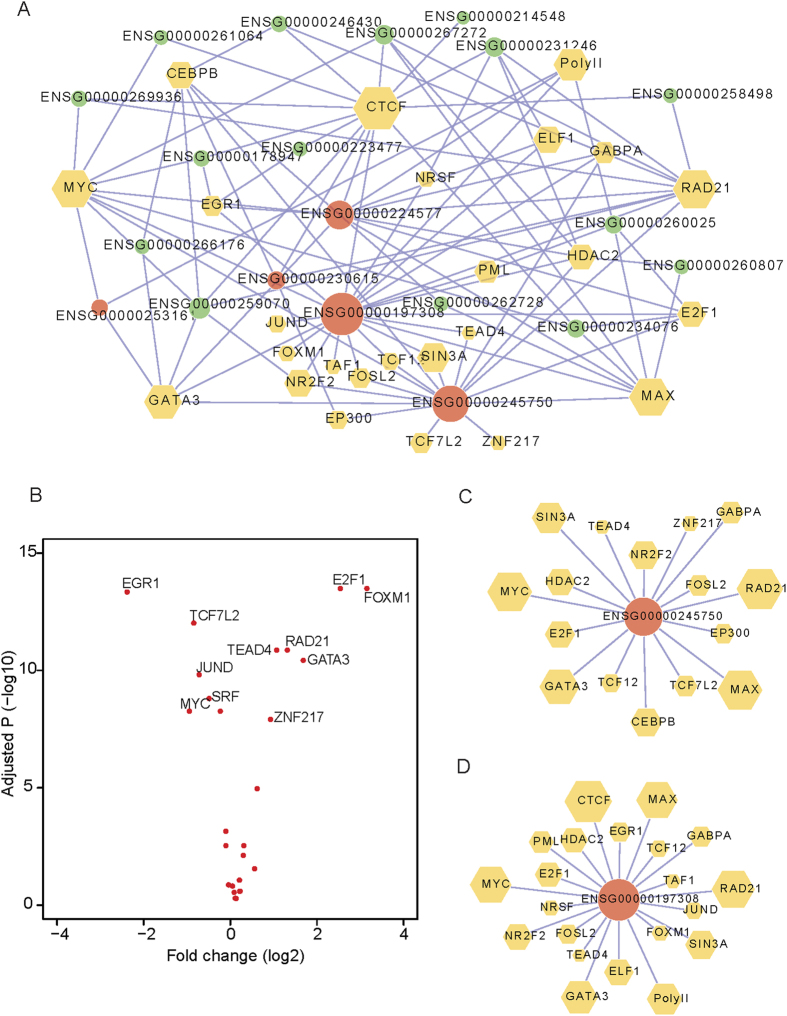
LincRNAs link to the regulation by transcription factors. (**A**) LincRNA-transcription factors (TFs) bipartite network. Each node with size scaled by its degree (the number of directly linked nodes) denotes one TF (yellow hexagon) or lincRNA (circle). Each edge represents the regulation of lincRNAs by TFs. Red and green circles represent the over-expressed and down-expressed lincRNAs in breast cancer samples, respectively. (**B**) Volcano plot of the log2-transformed fold change between breast cancer and adjacent normal tissues (X-axis, *n* = 85 pairs) versus –log10-transformed BH adjusted *P* value (Y-axis) for TFs (*n* = 26). The marked TFs show significantly differential expression in breast cancers relative to adjacent normal tissues. (**C,D**) Examples of two lincRNA genes (RP11-279F6 with Ensemble ID ENSG00000245750 and GATA3-AS1 with Ensemble ID ENSG00000197308) regulated by multiple TFs.

We then integrated 26 known TFs ChIP-seq data in MCF-7 cells, an ER+ breast cancer cell line from the ENCODE project (Table S4), to investigate whether and to what extent the 37 differentially expressed lincRNAs are functionally related to TFs. We constructed a lincRNA-TF bipartite network (see Methods), which consisted of 45 nodes and 101 edges (Fig. 2A). We found that 20 (54.1%) lincRNAs were bound by at least one TF, and 25 of the 26 TFs regulated at least one lincRNA. Especially, each of the eight key TFs, including GATA3, RAD21, MYC, CTCF, MAX, E2F1, CEBPB and RNA polymerase II, binds ≥ 5 lincRNAs. Among them, 11 TFs exhibited a significant differential expression (absolute log2-FC ≥ 0.585, BH-adjusted *P* < 0.01) in breast cancer tissues compared with adjacent normal tissues (Fig. 2B). We also found that several over-expressed lincRNAs may be related to these key TFs. For example, two lincRNA genes (RP11-279F6 with Ensemble ID ENSG00000245750 and GATA3-AS1 with Ensemble ID ENSG00000197308) had DNA binding peaks by 16 and 20 TFs, respectively. Specifically, all three over-expressed key regulators in breast tumors, GATA3, E2F1 and RAD21, showed connection with these two lincRNAs (Fig. 2C,D). These results suggest that the expression alteration of lincRNAs in breast cancer is probably functionally associated by one or multiple TFs in a complex way.

### Co-expression of lincRNAs with nearby protein-coding genes

**Figure 3:**
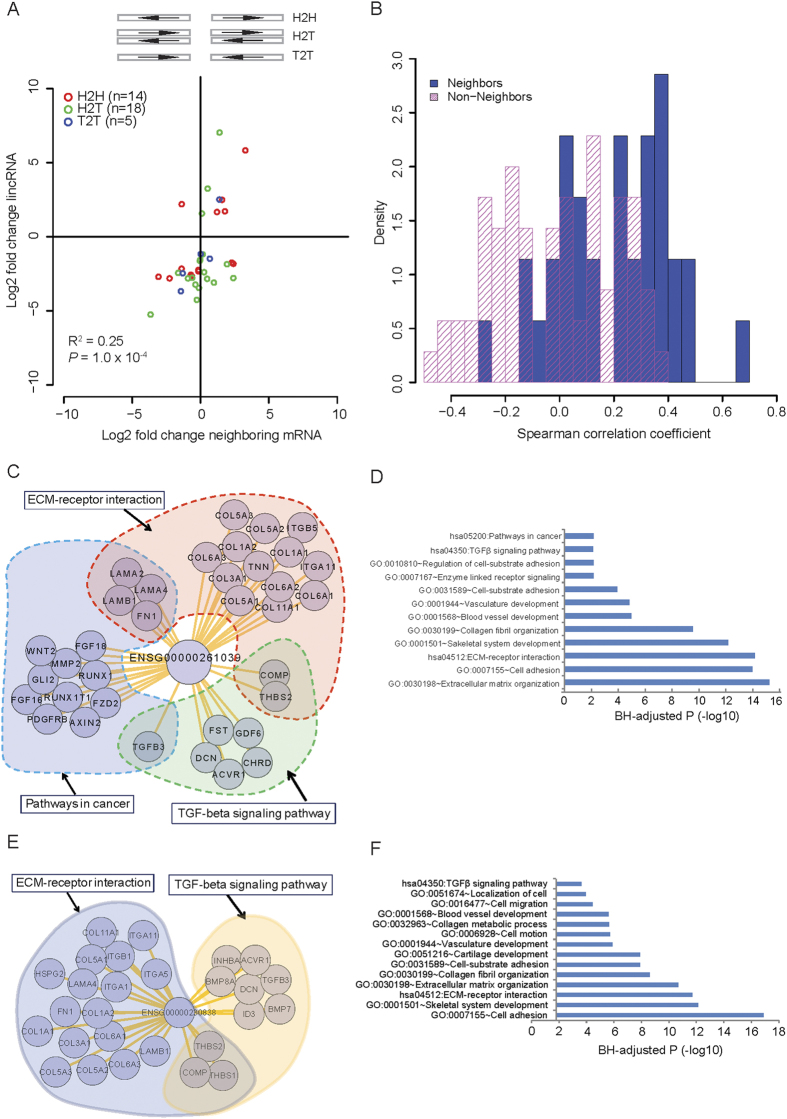
Co-expression differences between lincRNAs and neighboring mRNAs. (**A**) Plot of log2-transformed FC between 85 paired breast cancers for 37 lincRNAs (Y-axis) versus nearby mRNAs (X-axis). H2H, H2T and T2T represent the head-to-head, head-to-tail and tail-to-tail orientation between lincRNA and neighboring mRNAs, respectively (top panel). (**B**) Distribution of Spearman correlation coefficient from 85 adjacent normal tissues for lincRNA-neighboring pairs (blue) and lincRNA-non-neighboring pairs (pink). Functional enrichment and remarkable correlated protein-coding genes associated with lincRNA ENSG00000261039 (**C,D**) and ENSG00000230838 (**E,F**).

Several studies have reported that lincRNAs show an expression correlation with their neighboring protein-coding genes in normal tissues/cells^5^,^27^. We examined whether 37 DE lincRNAs could also exhibit the differential co-expression profile with the adjacent genes in breast cancer development. We classified these 37 lincRNA/mRNA pairs into three types: head-head (H2H), head-tail (H2T) and tail-tail (T2T) based on the paired transcriptional orientation (Fig. 3A). We observed a coordinated expression change (r^2^ = 0.25, *P* = 1.0 × 10^−4^) between lincRNAs and their neighbor mRNAs via a comparison of 85 paired breast tumor and adjacent normal breast tissues (Fig. 3A and B). We detected 14 (37.8%) lincRNA/mRNA pairs with the H2H type, which shows marginally significant over-representation (*p* = 0.06, one-sided Binomial test) relative to the global distribution. Similar results were observed when we analyzed 83 initially detected DE lincRNAs (Figure S3). Taking the H2H pair of *GATA3-AS1* (Ensembl ID ENSG00000197308) and its adjacent (aparting from 1220 bp) protein-coding gene *GATA3* as an example (Figure S4), the correlation coefficient between these two genes was 0.54 with *P* = 6.7 × 10^−5^. These observations are consistent with the previous reports that both *GATA3-AS1* and *GATA3* are co-expressed in mouse and human TH2 cells^28^,^29^. Altogether, the lincRNAs divergently transcribed with protein-coding genes are more likely to show the differentially co-expression profiles.

To further determine the uniqueness of the differential co-expression observation of lincRNA-mRNA adjacent pairs, we characterized the co-expression patterns of non-neighboring lincRNA-mRNA pairs and randomly shuffled lincRNA-mRNA pairs using the same 85 pairs of breast cancer/adjacent normal tissue in discovery stage (see Methods). As expected, there were no co-expression changes for the random pairs (Figure S5). In addition, the neighboring lincRNA-mRNA pairs showed a higher Spearman’s rank correlation coefficient than those of non-neighboring lincRNA-mRNA pairs (Fig. 3B).

### Functional Prediction of lincRNAs

To date, thousands of lincRNAs have been annotated, while the biological functions are unclear for most of them. Based on the Refseq genes showing strong co-expression relationship with lincRNAs, a method commonly used for functional prediction of unknown genes^30^,^31^, we predicted the biological functions for these 37 lincRNAs (see Methods). Enrichment analysis of GO terms and KEGG pathways showed that these down-regulated lincRNAs might be associated with transcriptional regulation, RNA processing and translational elongation processes, *etc*. In contrast, the up-regulated lincRNAs probably participate in cancer cell migration and proliferation, including cell adhesion, regulation of epithelial cell proliferation and regulation of cell cycle (Table S5). For example, two over-expressed lincRNAs (*RP11-417E7* with Ensemble ID ENSG00000261039 and *AC093850* with Ensemble ID ENSG00000230838) showed over-expression in both the discovery and the replication stage. These two lincRNAs may be involved in the ECM-receptor interaction and cell adhesion, TGFβ signaling pathway and others (Fig. 3C–F). The TGFβ signaling pathway is well documented with a promoter of tumor progression and invasion^32^. Together, these two lincRNAs probably participate in the pathogenesis of breast cancer.

### LincRNAs associated with breast cancer subtypes

**Figure 4:**
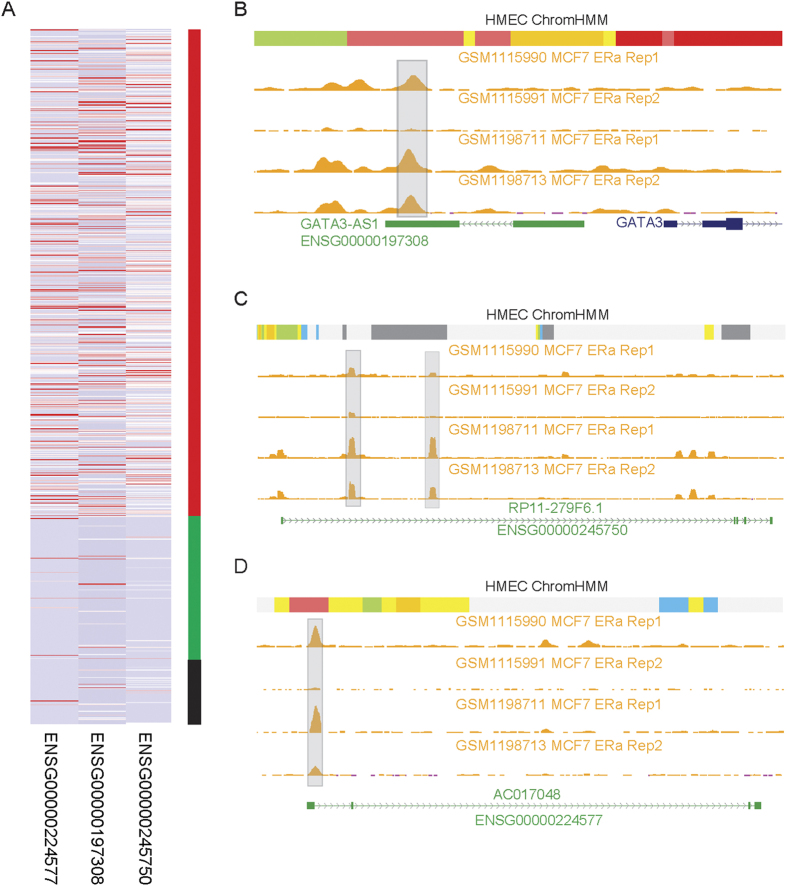
Specific expression of lincRNAs in breast cancer subtypes. (**A**) Heatmap of three lincRNAs specifically over-expressed in ER+ breast cancer. Red and green represent 664 ER+ and 196 ER- cancer samples from TCGA, respectively. Black bar denotes 85 adjacent normal tissues. Distribution of DNA binding by ERα in three lincRNA genes, (**B**) *GATA3-AS1* (Ensemble ID ENSG00000197308), (**C**) *RP11-279F6* (Ensemble ID ENSG00000245750) and (**D**) *AC017048* (Ensemble ID ENSG00000224577). The gray bars represent the DNA binding enrichment for the ERα in the MCF-7 cells. The track in the top for each lincRNA is the chromatin states from the chromHMM algorithm in the HMEC cell line. Chromatin states with bright red and light red, orange and yellow, blue, green and grey represent active promoter and weak promoter, strong enhancer and weak/poised enhancer, insulator, transcriptional region and heterochromatin/low signal, respectively.

We also investigated whether these 37 DE lincRNAs exhibited expression difference across different breast cancer subtypes. We found three lincRNAs: GATA3-AS1 (ENSG00000197308), RP11-279F6 (ENSG00000245750) and AC017048 (ENSG00000224577), showed specifically high expression levels in ER-positive (ER+), compared to ER-negative (ER-) cancers and normal breast tissue samples (Fig. 4A). The specific expression alteration of these three lincRNAs in ER+ subtype was also validated in the replication stage. We also used the data of differentially expressed lincRNAs across four breast cancer subtypes (Luminal A, Lumnal B, Her2, and Basal-like) reported in Su *et al*. study^30^ to evaluate the subtype associations for these 37 lincRNAs. We found that the 22 (59.5%) lincRNAs (8 up-regulated lincRNAs and 14 down-regulated lincRNAs) were differentially expressed in at least one breast cancer subtype (Table S6). Three above reported lincRNAs over-expressed in the ER+ subtype (Fig. 4A) showed much higher expression levels in *Luminal A* and *Lumnal B* subtypes (enriched for ER+), relative to *Her2, and Basal-like* subtypes.

Further integrating two independent estrogen receptor alpha (ERα) ChIP-seq dataset (each with two replicates) in MCF-7 cancer cells, we observed the ERα binding sites in all three lincRNA loci (Fig. 4B–D). For example, an ERα DNA binding site was found near the transcriptional terminal region of the GATA3-AS1 based on the analysis of ERα ChIP-seq data (Fig. 4B). This binding region was further annotated as the active enhancer region using chromatin states in human mammary epithelial cells (HMECs). Similarly, this enhancer state was also observed in the other two lincRNAs (Fig. 4C,D). These results indicated that these three ER status associated lincRNAs may be regulated by ERα.

### LincRNAs associated with breast cancer survival

**Figure 5:**
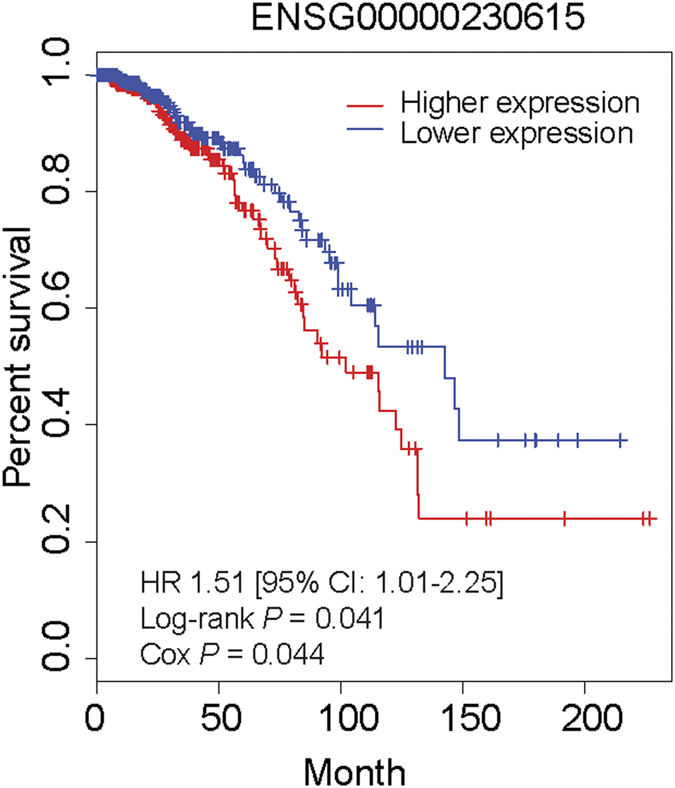
Association of lincRNA expression levels with breast cancer survival. Kaplan-Meier survival plots for the lincRNA RP5-1198O20 (Ensemble ID ENSG00000230615). Two groups (higher and lower expression) are separated on the basis of the median expression level of lincRNAs across breast cancer patients.

We assessed the association of those 37 lincRNAs expression levels with breast cancer survival and found one candidate (RP5-1198O20 with Ensembl ID ENSG00000230615, Fig. 5). Patients with expression levels of lincRNA ENSG00000230615 higher than the median value (median FPKM value is 2.8) had worse survival rates than those women with expression levels less than the median value (median FPKM value is 0.5) (Fig. 5).

## Discussion

In this study, we analyzed lincRNA transcriptome in over 1000 breast tissue samples. Two independent sequencing datasets consistently identified a set of lincRNAs deregulated in breast carcinogenesis. Importantly, the protein-coding genes neighboring these deregulated lincRNA loci also showed expression alternation in breast cancer tissues, implying the transcriptional concordance between lincRNAs loci and neighboring genomic regions in cancer development. The expression aberration of lincRNAs in breast cancer may be associated with the expression alteration of multiple TFs. Our work substantially extends the biological understanding of the lincRNA repertoire in the pathogenesis of breast cancer.

It should be noted that the aberrant expression of several lincRNAs previously identified in multiple cancers showed deregulation in the present study. For example, in both discovery and validation stages, expression levels of the *MEG3* decreased in breast tumor samples, which is consistent with the proposed tumor suppressor role for *MEG3*^12^,^30^. Another un-regulated lincRNA, *GATA3-AS1* (Ensembl ID ENSG00000197308) was also identified in Ding *et al*. study^33^. Our *in silico* functional prediction (Table S5) indicated the *GATA3-AS1* probably performs an immune response associated role in breast cancer progression. This result was supported by previous two studies^29^,^34^ showing that the *GATA3-AS1* is highly expressed in T helper subsets. Spurlock *et al*. also speculated that the *GATA3-AS1* might play a role in allergic or asthmatic responses^34^. In addition, this lincRNA was also reported to have a decreased expression level in brain, bladder and prostate cancers^22^. Another breast cancer survival associated lincRNA *RP5-1198O20* (Ensembl ID ENSG00000230615) showed the up-regulation in both stages of this study. Re-examining Gibb *et al*. integrated SAGE-seq findings^22^, we confirmed that this lincRNA also showed an increased expression in breast cancers. Another study about transcriptome analysis of aging identified the down-regulation of this lincRNA^35^. Further functional investigation of the lincRNA *RP5-1198O20* in either carcinogenesis or aging would be interesting. In another example, our data showed the down-regulation of the miRNA-145 host gene (MIR145 with Ensembl ID ENSG00000269936) in breast cancer, consistent with previous reports in breast cancer^36^ and other cancer types^37^,^38^. However, several well-characterized lincRNAs^12^,^22^,^23^,^39^,^40^, including *HOTAIR, H19, GAS5, PCA3, PVT1*, were not investigated in this study. Those lncRNAs are not transcribed from intergenic regions, belonging to either anti-sense or other types of long non-coding transcripts, according to the GENCODE (version17) annotation. That means these lncRNAs are difficult to distinguish from their host protein-coding genes using the typical RNA-seq technology. There are several lncRNAs with no polyA tails^41^,^42^ which are unmeasured in the current study as well. Therefore, more sophisticated methods, such as strand-specific and non-poly(A) tail RNA-seq technology, are required to distinguish anti-sense transcripts from protein-coding genes, and to comprehensively capture the lncRNA transcriptome.

Several lincRNAs show expression alteration in other cancers, including *NEAT1* (Ensembl ID ENSG00000245532) down-regulated in retinoblastoma^22^, *MALAT1* (Ensembl ID ENSG00000251562) up-regulated in lung and colorectal cancer^43^,^44^. However, these lincRNAs do not exhibit expression aberration in breast cancer. Likewise, the *PCAT1* (Ensembl ID ENSG00000253438), a prostate-specific lincRNA regulating cell proliferation, shows over-expression in a subset of prostate cancers^45^. In our study, we almost did not detect the expression level of the *PCAT1* in either breast normal or breast tumor samples. These results partially support the observation that many lincRNAs are expressed in a tissue- and cancer-type restricted manner, making them useful as prognostic markers^12^.

However, some limitations remain in this study. Firstly, no functional validations on these DE lincRNAs prevent us draw the further conclusion about how the aberrant expression of lincRNAs contribute to tumorigenesis. Secondly, the relative small sample size and the unavailability of breast cancer subtype data in the replication stage also prevent us replicate the subtype-specific lincRNAs. Finally, due to the tumor heterogeneity and the cell mixture in tumor tissues, global comparison of gene expression profiles among breast tumor, adjacent and normal tissues is insufficient, particularly for lincRNAs whose expression patterns broadly show the cell- or tissue-type specificity. Thus, single-cell or sorted cell population based transcriptomic analysis^46^,^47^ will be favorable to determine these lincRNAs as robust biomarkers.

## Conclusions

We identified a signature of lincRNA expression profile for breast cancer. Further functional surveys of these lincRNAs will be warranted to discover the biological mechanisms of lincRNA in breast cancer development and progression.

## Materials and Methods

### Discovery dataset

After approval, raw RNA-seq data (Level 1) of breast tumor tissues (N = 915) and adjacent normal breast tissues (N = 85) were acquired from the TCGA. Clinical data (Biotab format) for these 915 breast invasive carcinoma (BRCA) were also acquired from the TCGA.

### Replication dataset

Our replication study consisted of 50 breast cancer cases and 23 healthy controls. The patients were pathologically confirmed for primary breast cancer diagnosed at one of three hospitals in Indianapolis, Indiana, between 1998 and 2009: Indiana University Hospital, Wishard Hospital (now Eskenazi Hospital), and the Indiana University Simon Cancer Center (IUSCC). Controls were randomly selected from a pool of healthy women who donated normal breast tissues to the Susan G. Komen Tissue Bank between 2005 and 2009, and were free of breast cancer at the time of donation. The participants completed a questionnaire on medical histories and health-related exposures at the time of donation. Controls were frequency matched to cases based on self-reported ancestry and age (within 5 years).

Breast tissue (untreated tumor or normal) biospecimens were collected from each case and control under standard operating procedures at IUSCC. All breast tissue samples were snap-frozen immediately after removal and stored in liquid nitrogen until processed, and were determined to be of high quality through histological and molecular quality control tests. Tumor samples were pathologically verified for high tumor content. Information concerning demographics, clinical data, and personal risk factors, including age and race, were either extracted from medical records (for cases) or obtained through the questionnaires completed by the participants (for controls).

Total RNA was extracted from freshly frozen breast tissue samples using the Qiagen^®^ miRNeasy Mini Kit. Construction of cDNA libraries and subsequent RNA sequencing of paired-end reads (2 × 50 nt reads) were performed according to the standard Illumina protocol using the HiSeq2000 sequencing systems. The raw sequencing output was 25–35 million reads per sample.

### Other dataset

The annotation data for known lincRNAs (n = 6,020) were extracted from the gencode.v17.long_noncoding_RNAs.gtf.gz file from the GENCODE database. Other data used in this study include: human Refseq genes obtained from the NCBI database; protein-DNA interactions from ChIP-seq data in MCF-7 breast cancer cell lines downloaded from ENCODE project^48^; DNA binding by ERα using ChIP-seq in MCF-7 cells, and RNA-seq data across 14 breast cancer cell lines^49^,^50^ downloaded from the Gene Expression Omnibus (GEO) database^51^.

### RNA-seq data processing and lincRNA annotation

In the discovery stage, for the 85 pair of matched breast tumor and normal tissues, mapped reads in BAM format (Level 1) were assembled with Cufflinks (v2.1.1)^52^. The lincRNA annotation was conducted by the following procedures: 1) we retained assembled transcripts whose genomic loci are overlapped or imbedded with known annotated lincRNAs; 2) we removed transcripts in length of <200 nt; 3) we eliminated transcripts showing coding potential (score <0.5) predicted with iSeeRNA program (version 1.2)^53^; 4) if two or more transcripts (isoforms) mapped to a lincRNA locus, we assigned the mean coverage and expression value to that lincRNA. We applied the same procedures for the remaining RNA-seq data, except for publically accessible RNA-seq data that were processed as described elsewhere^54^. To reduce the noise caused by the lincRNA expression variability between samples, we plotted the calling rate (occurrence of lincRNA transcription based on Cufflinks FPKM ≥ 0.3) versus lincRNAs ranked by missing rate across 170 samples. As Ramskold *et al*. proposed^55^, this cutoff (FPKM ≥ 0.3) is an optimized threshold for detectable expression above background. Then, we set the calling rate ≥ 20% as a threshold to ensure high-confidence lincRNAs for further analyses.

For RNA-seq data in the replication stage, quality control (QC) filtering was first performed on raw RNA-seq data to remove adapter sequences and poor quality bases using the FastqMCF clipping algorithm. RNA-seq reads were then mapped by Bowtie v1.0.0, to GENCODE lncRNA reference (release 17) for lncRNA (including lincRNA) annotations. Transcript abundances were quantified using NGSUtils. Samples were further filtered based on percentage of genes detected (less than 50%) and percentage of reads mapped to the reference (less than 25%). Extreme outliers were further identified and filtered from the dataset using principal component analysis (PCA). After these steps, a total of 7,450 lncRNAs retained and were used in further analyses.

### ChIP-seq data processing

For ChIP-seq data from the ENCODE project, we directly downloaded files with called peaks for subsequent analyses. For two independent ERα ChIP-seq data whose processed peaks were unavailable, we downloaded the raw data in FASTQ format and conducted the peak calling as follows. Reads were mapped to the human reference genome (hg19) using Bowtie2 program^56^ in the default parameters. Aligned data were processed and converted into BAM files using SAMtools program^57^. Then, we used the MACS14 (version 1.4.2) program^58^ to call peaks in 20 bp resolution. We visualized the results in the UCSC Genome Browser.

### Differential expression of lincRNAs

The fragments per kilobase of exon per million fragments mapped (FPKM) values were calculated from the Cufflinks program to represent lincRNA expression levels. The differential expression of each lincRNA between breast tumor and adjacent normal tissues was defined as: fold change (FC) ≥ 2 with Benjamini-Hochberg (BH) adjusted *P* < 0.01 based on non-parametric Wilcoxon rank sum paired test.

Following the permutation-based method developed by Xie *et al*.^59^, we estimated the FDR in identifying lincRNAs with differential expression. Briefly, FDR is estimated as 
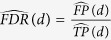
, where *d* is a fixed threshold, 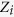 is a test statistic and *B* = 1000 permutations. 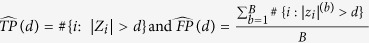. The 1000 permutations were achieved by shuffling the tumor/normal samples in the lincRNA-sample matrix (584 lincRNAs and 170 samples).

For RNA-seq data in the replications stage, differential expression (DE) analyses were performed using edgeR v2.6.12, implemented in the Bioconductor package to identify differentially expressed lncRNAs between tumor and adjacent normal, as well as tumor and normal breast tissue. The trimmed mean of M-values (TMM) method was used to normalize the raw read counts. Biological coefficients of variation between the samples were estimated using an empirical Bayes approach under the assumption that the data follows a negative binomial distribution. Differential expression between tumor and adjacent normal or normal breast tissue was analyzed using a generalized linear model to regress lncRNA (expression on tissue type, adjusting for age, race, and sequencing batch). Statistical significance was defined as FDR p-value < 0.05 and a two-fold change (FC) of expression level between comparison of tumor and adjacent normal or normal breast tissue.

### Construction of transcription factor and lincRNA network

We downloaded ChIP-seq peak files of 26 transcription factors (TFs) in the MCF-7 breast cancer cells from the ENCODE project. A lincRNA bound by a TF was determined, if the TF binding peak is located within the region of 2 kb upstream of its transcription start site (TSS) to transcription end site (TES) of the lincRNA locus. Then, a lincRNA-TF bipartite network was constructed using the Cytoscape (v2.8.3) software^60^, where each node denotes a lincRNA or TF and each edge represents a lincRNA-TF interaction. The degree of each node in this bipartite network was calculated. We regarded the nodes representing TFs having ≥ 5 degrees as key TFs.

### Co-expression analysis

We used similar methods for lincRNA annotation to annotate Refseq protein-coding genes and calculate their FPKM values. The neighboring protein-coding genes were defined as the ones with the closest physical distance to known lincRNAs. We coupled lincRNA-encoding locus and neighboring protein-coding genes, and referred to it as a lincRNAs-mRNA pair. We further classified lincRNAs-mRNA pairs into three types, according to their transcriptional orientation: head-to-head (H2H), head-to-tail (H2T) and tail-to-tail (T2T). The linear relationship of log2-transformed fold change in a comparison of 85 paired samples for lincRNA-mRNA pairs was used to evaluate the coordinated changes.

We defined the non-neighboring genes as the ones over a 1 Mb physical distance from lincRNAs on both strands. We coupled them as non-neighboring lincRNAs-mRNA pairs. Similarly, we determined the coordinated changes for non-neighboring lincRNA/mRNA pairs. We repeated the analysis using randomly selected 1,000 lincRNA-mRNA pairs (regardless of the distance) from the entire transcriptome data.

### Functional prediction of lincRNAs

To predict biological functions for these DE lincRNAs, we calculated the correlation coefficients between DE lincRNAs and all Refseq protein-coding genes using the Spearman rank correlation analysis. We regarded the Spearman rank correlation coefficients calculated from randomly shuffling lincRNA/mRNA pairs (1000 times) as null distribution. Compared with the null distribution, we set a threshold for the Spearman rank correlation coefficient ≥ 0.4 (or ≤ −0.4) (Figure S6) to reflect the strong co-expression between lincRNAs and Refseq genes in high confidence. On this basis, a set of Refseq genes passing this threshold were regarded as the functional association to lincRNAs, and used for functional enrichment analysis using DAVID annotation^61^. GO terms with BH-adjusted *P* ≤ 0.05 served as functional enrichment for lincRNAs.

### Survival analyses

Excluding participants with unknown survival information (*n* = 16), the remaining 899 subjects were retained in survival analyses. We split patients into two groups (higher and lower expressions of lincRNA) based on the median level of lincRNA expression. The Kaplan-Meier curve and hazard ratio (HR) of higher versus lower expressed groups were generated in R (versions 2.15.0) using the *survival* package.

### Ethical consent

Utilization of data was conducted in accordance with TCGA data access policies. Signed informed consent was obtained from each subject in the replication study. The study was approved by Indiana University institutional review board.

## Additional Information

**How to cite this article**: Zhang, Y. *et al*. Long intergenic non-coding RNA expression signature in human breast cancer. *Sci. Rep.* **6**, 37821; doi: 10.1038/srep37821 (2016).

**Publisher's note:** Springer Nature remains neutral with regard to jurisdictional claims in published maps and institutional affiliations.

## Supplementary Material

Supplementary Information

## Figures and Tables

**Table 1:** Clinical characteristic of study subjects in this study.

	Discovery stage		Replication stage	
	Paired tumor-normal	Only tumor tissues^b^	Paired tumor-normal tissues	Healthy Women
**No. of samples**	85	830	50	23
**Age (yr ± s.d.)**	57.2 ± 15.4	58.4 ± 12.9	51.7 ± 10.9	52.0 ± 11.3
Ethnicity				
White	79 (92.9%)	588 (70.8%)	39 (78%)	18 (78%)
Asian	1 (1.2%)	56 (6.8%)	0	0
Black	5 (5.9%)	79 (9.5%)	11 (22%)	5 (22%)
Unknown	0	107 (12.9%)	0	0
ER status				
ER+	67 (78.9%)	597 (71.9%)	40 (80%)	NA
ER^−^	15 (17.6%)	181 (21.8%)	10 (20%)	NA
Unknown	3 (3.5%)	52 (6.3%)	0	NA
Stage				
0-I	16 (18.8%)	135 (16.2%)	2 (4%)	NA
II	46 (54.1%)	469 (56.5%)	14 (28%)	NA
III	20 (23.5%)	179 (21.6%)	6 (12%)	NA
IV	2 (2.4%)	13 (1.6%)	1 (2%)	NA
Unknown	1 (1.2%)	34 (4.1%)	27 (54%)	NA

^a^BRCA (breast invasive carcinoma) with matched adjacent normal tissues.

^b^BRCA without matched adjacent normal tissues.

**Table 2:** Expression alteration of 37 lincRNAs in discovery and replication stages.

Ensembl ID	Symbol	Discovery stage^a^		Replication Stage^b^		Replication Stage^c^	
		Log2-FC^d^	*P*	Log2-FC^d^	*P*	Log2-FC^d^	*P*
ENSG00000261039	RP11-417E7	7.04	1.47E-08	3.49	2.42E-03	7.11	6.45E-05
ENSG00000230838	AC093850	5.84	6.87E-08	9.72	1.19E-10	15.03	1.42E-11
ENSG00000224577	AC017048	3.26	4.31E-05	2.17	2.69E-02	1.03	4.56E-01
ENSG00000253364	RP11-731F5	2.52	4.88E-03	9.31	1.78E-09	13.17	1.16E-08
ENSG00000197308	GATA3-AS1	2.49	2.08E-05	8.27	6.36E-07	5.58	1.43E-02
ENSG00000259093	RP11-1112J20	2.20	6.87E-08	2.02	8.65E-07	2.78	4.02E-06
ENSG00000230615	RP5-1198O20	1.72	2.80E-04	6.53	1.75E-13	7.58	3.50E-09
ENSG00000253161	RP11-150O12	1.68	1.00E-03	4.61	4.12E-05	5.54	9.67E-04
ENSG00000245750	RP11-279F6	1.57	1.17E-03	4.96	1.63E-04	6.07	1.47E-03
ENSG00000214548	MEG3	−1.17	2.47E-07	−2.35	1.15E-07	−0.75	2.18E-01
ENSG00000237697	LINC00312	−1.18	9.13E-03	−3.10	1.24E-04	0.41	7.27E-01
ENSG00000267519	CTD-3252C9	−1.48	6.89E-11	−1.01	4.12E-05	1.33	2.00E-05
ENSG00000267272	RP5-1052I5	−1.56	2.64E-05	−3.02	2.65E-04	−2.37	3.67E-02
ENSG00000255455	RP11-890B15	−1.62	1.28E-04	−1.59	3.60E-04	−1.35	3.52E-02
ENSG00000234076	TPRG1-AS1	−1.74	3.52E-03	−1.93	1.12E-03	−3.37	2.06E-05
ENSG00000262728	AC123768	−1.82	4.90E-08	−1.58	1.27E-02	−3.01	2.80E-04
ENSG00000269930	RP11-932O9	−1.85	9.95E-07	−1.82	8.56E-05	−1.74	7.45E-03
ENSG00000260025	RP11-490M8	−2.15	1.10E-06	−1.99	1.18E-02	−2.78	6.05E-03
ENSG00000260807	RP11-161M6	−2.24	1.64E-06	−1.96	4.49E-02	−5.65	1.34E-05
ENSG00000231246	RP5-965F6	−2.32	4.87E-07	−3.42	5.22E-03	−2.94	7.71E-02
ENSG00000266176	RP11-855A2	−2.39	9.44E-04	−4.16	9.90E-05	0.16	9.21E-01
ENSG00000260693	AC026150	−2.44	1.08E-06	−1.81	3.04E-04	−1.19	8.99E-02
ENSG00000223477	LINC00842	−2.46	8.86E-08	−2.93	7.21E-04	−5.50	1.07E-06
ENSG00000182021	RP11-381O7	−2.57	2.33E-05	−3.26	1.17E-06	−3.67	8.14E-05
ENSG00000258498	DIO3OS	−2.68	1.01E-04	−3.34	2.49E-05	−3.76	9.62E-05
ENSG00000269936	MIR145	−2.70	9.87E-13	−1.34	5.52E-04	−1.61	1.80E-03
ENSG00000229645	LINC00341	−2.77	6.89E-11	−3.89	7.12E-06	−2.82	1.50E-02
ENSG00000261064	RP11-1000B6	−2.79	9.95E-07	−1.33	7.23E-03	−1.77	6.81E-03
ENSG00000229108	AC005550	−2.81	2.28E-05	−4.31	4.11E-04	−6.34	7.85E-05
ENSG00000267194	RP1-193H18	−2.81	2.97E-06	−2.32	1.43E-03	−3.05	7.26E-04
ENSG00000260124	RP4-791K14	−2.84	8.18E-06	−1.84	8.56E-06	−2.32	4.72E-05
ENSG00000224307	RP11-344B5	−3.10	7.24E-04	−2.07	2.87E-03	−1.88	3.44E-02
ENSG00000178947	LINC00086	−3.23	1.94E-09	−1.59	2.60E-02	−2.66	5.14E-03
ENSG00000259070	LINC00639	−3.45	2.88E-07	−2.30	8.87E-04	−3.48	1.40E-04
ENSG00000271239	RP11-238F2	−3.67	3.37E-06	−3.19	3.08E-02	−3.86	5.24E-02
ENSG00000262179	RP1-302G2	−4.26	6.95E-07	−5.21	3.39E-06	−4.74	1.56E-03
ENSG00000246430	RP11-16M8	−5.25	2.61E-06	−6.36	4.27E-09	−3.19	3.97E-02

^a^The discovery stage from TCGA.

^b,c^The replication stage from an independent study; ^b^comparison between paired tumor and adjacent normal breast tissue from cancer patients; ^c^comparison between tumor and normal breast tissue from healthy controls.

^d^Log2-FC denotes log2-transformed fold change between breast tumor and non-tumor samples.
