# Supplementary material for: Long intergenic non-coding RNA expression signature in human breast cancer
Source: Sci Rep. 2016 Nov 29;6:37821. doi: 10.1038/srep37821 (PMC5126689; doi:10.1038/srep37821)
Supplement: Supplementary Information [file srep37821-s1.pdf]

Long intergenic non-coding RNA expression signature in human breast cancer

Yanfeng Zhang[1,5,#], Erin K. Wagner[2,#], Xingyi Guo[1], Isaac May[3], Qiuyin Cai[1], Wei Zheng[1], Chunyan He[2,4,*], Jirong Long[1,*]

[1] Division of Epidemiology, Department of Medicine, Vanderbilt University Medical Center, Nashville, TN 37203, USA, youngorchuang@hotmail.com, xingyi.guo@vanderbilt.edu, qiuyin.cai@vanderbilt.edu, wei.zheng@vanderbilt.edu

[2] Department of Epidemiology, Richard M. Fairbanks School of Public Health, Indiana University, Indianapolis, IN 46202, USA, chunhe@iu.edu

[3] Bowdoin College, Brunswick, ME, 04011

[4] Indiana University Melvin and Bren Simon Cancer Center, Indianapolis, IN 46202, USA

[5] Present address: HudsonAlpha Institute for Biotechnology, Huntsville, AL 35806, USA

[#] Contributed equally

***Correspondence to:**

Jirong Long, Ph.D.

Vanderbilt Epidemiology Center and Vanderbilt-Ingram Cancer Center

Vanderbilt University Medical Center

2525 West End Avenue, 8th Floor, Nashville, TN 37203

Phone: (615) 343-6741; Fax: (615) 936-8241

E-mail: Jirong.Long@vanderbilt.edu

Chunyan He, Sc.D.

Department of Epidemiology

Indiana University Richard M. Fairbanks School of Public Health

Indiana University Melvin and Bren Simon Cancer Center

980 W. Walnut Street, R3, C241

Indianapolis, IN 46202

Phone: (317) 278-3033; Fax: (317) 278-2966

E-mail:chunhe@iu.edu

Supplementary Information

Table *S1*: Summary of meta-data, including ChIP-seq data for transcription factor ERα and RNA-seq data used in this study.

Table *S2*: LincRNAs validated in either tumor-adjacent normal paired samples or tumor-normal health women.

Table *S3*: Expression levels of 37 lincRNAs across 14 breast cancer cell lines.

Table *S4:* Expression alteration of 26 transcription factors in 85 pairs of breast cancer and adjacent normal tissue.

Table *S5*: Functional prediction of 37 lincRNAs in both tumor and adjacent normal tissues.

Table S6: Expression levels of 22 lincRNAs showing differential expression across breast cancer subtypes.

Figure *S1*: plot of the calling rate of lincRNAs versus the number of lincRNAs not detected. The horizontal dashed line represents the threshold used in this study.

Figure *S2*: volcano plot of the log2-transformed fold change between breast cancer tissues and adjacent normal tissues (X-axis, n = 85 pairs) versus –log10-transformed BH adjusted P value (Y-axis) for lincRNAs (n = 584).

Figure S3: (A) plot of log2-transformed FC between 85 paired breast cancers for 83 lincRNAs (Y-axis) versus nearby mRNAs (X-axis). H2H, H2T and T2T represent the head-to-head, head-to-tail and tail-to-tail orientation between lincRNA and neighboring mRNAs, respectively. (B) Distribution of Spearman correlation coefficient from 85 adjacent normal tissues for lincRNA-neighboring pairs (blue) and lincRNA-non-neighboring pairs (purple).

Figure *S4*: plot of the expression correlation between the lincRNA GATA3-AS1 and its adjacent protein-coding gene GATA3.

Figure *S5*: (A) plot of fold change (log2-tranformed) of 37 lincRNAs (Y-axis) versus non-neighboring mRNAs (X-axis) between 85 pairs of breast cancer and adjacent normal tissues. (B) Plot of fold change (log2-tranformed) of 584 lincRNAs (Y-axis) versus randomly selected mRNAs (X-axis) between 85 pairs of breast cancer and adjacent normal tissues.

Figure *S6*: density plot of Spearman rank correlation coefficient presenting the co-expression profiling between lincRNAs and mRNAs in breast cancer and adjacent normal tissues, as well as in a null distribution.

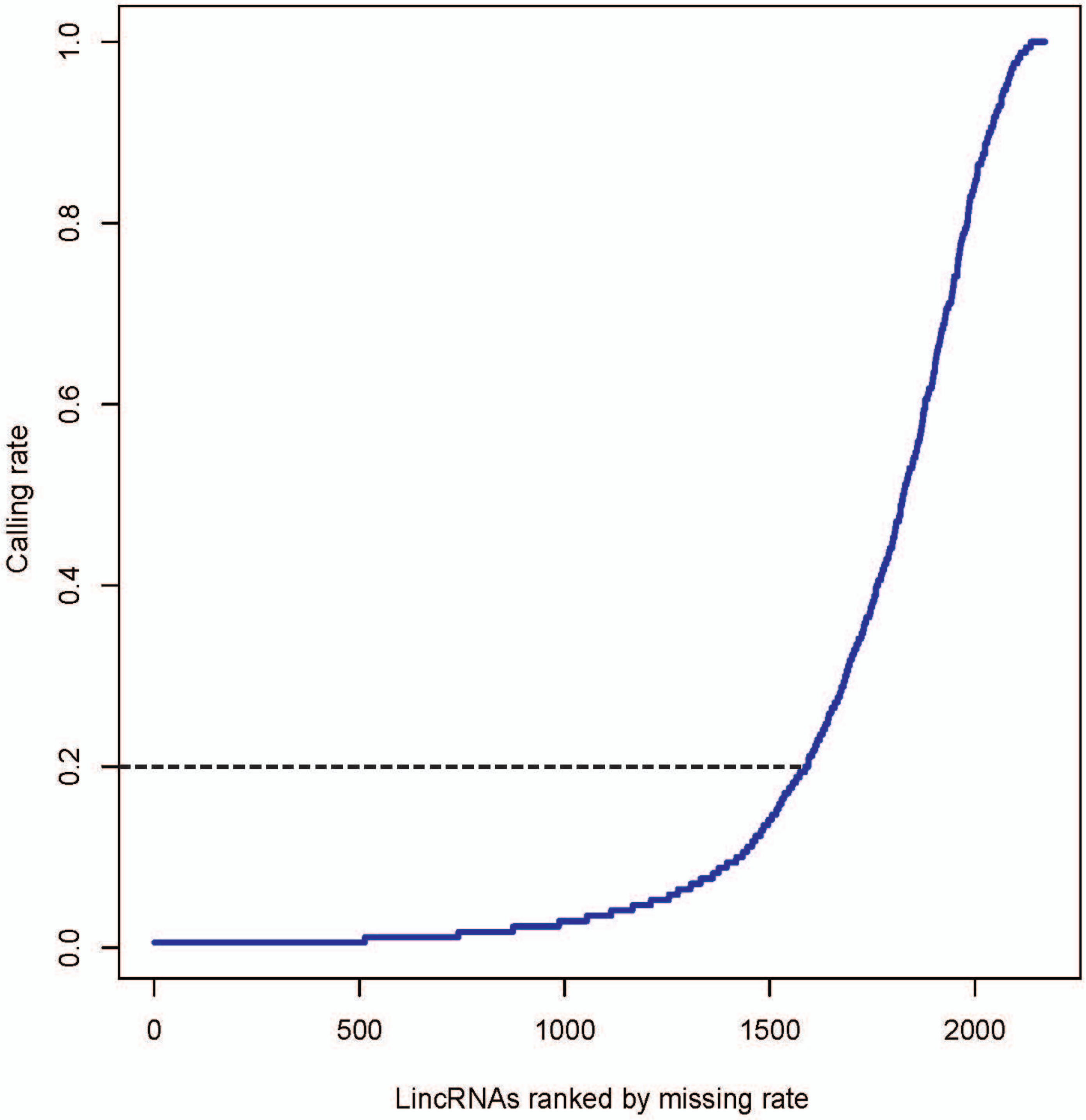

Calling rate
1.0
0.8
0.6
0.4
0.2
0.0
0
500
1000
1500
2000
LincRNAs ranked by missing rate

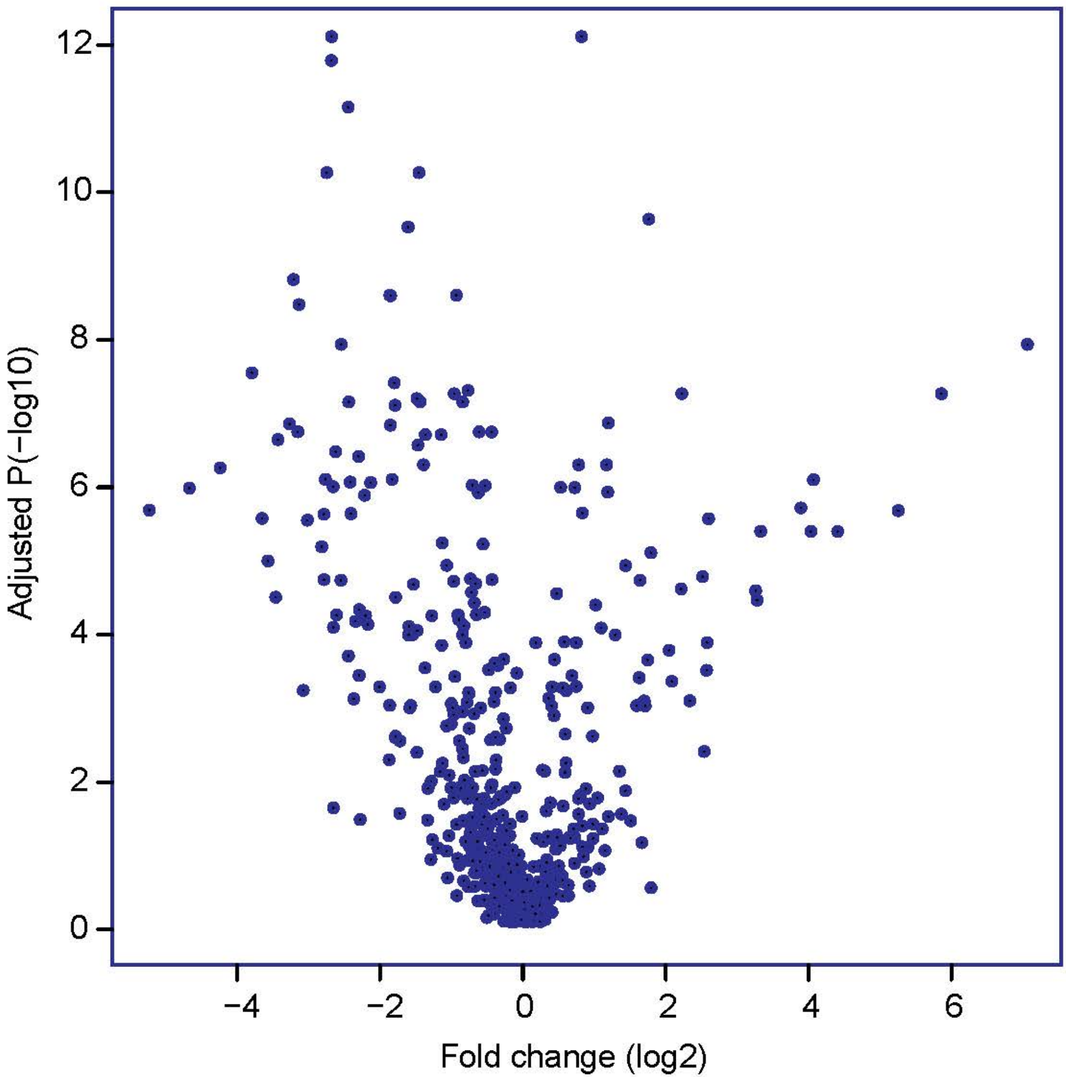
12
10
8
6
4
2
0
Adjusted P(−log10)
−4
−2
0
2
4
6
Fold change (log2)

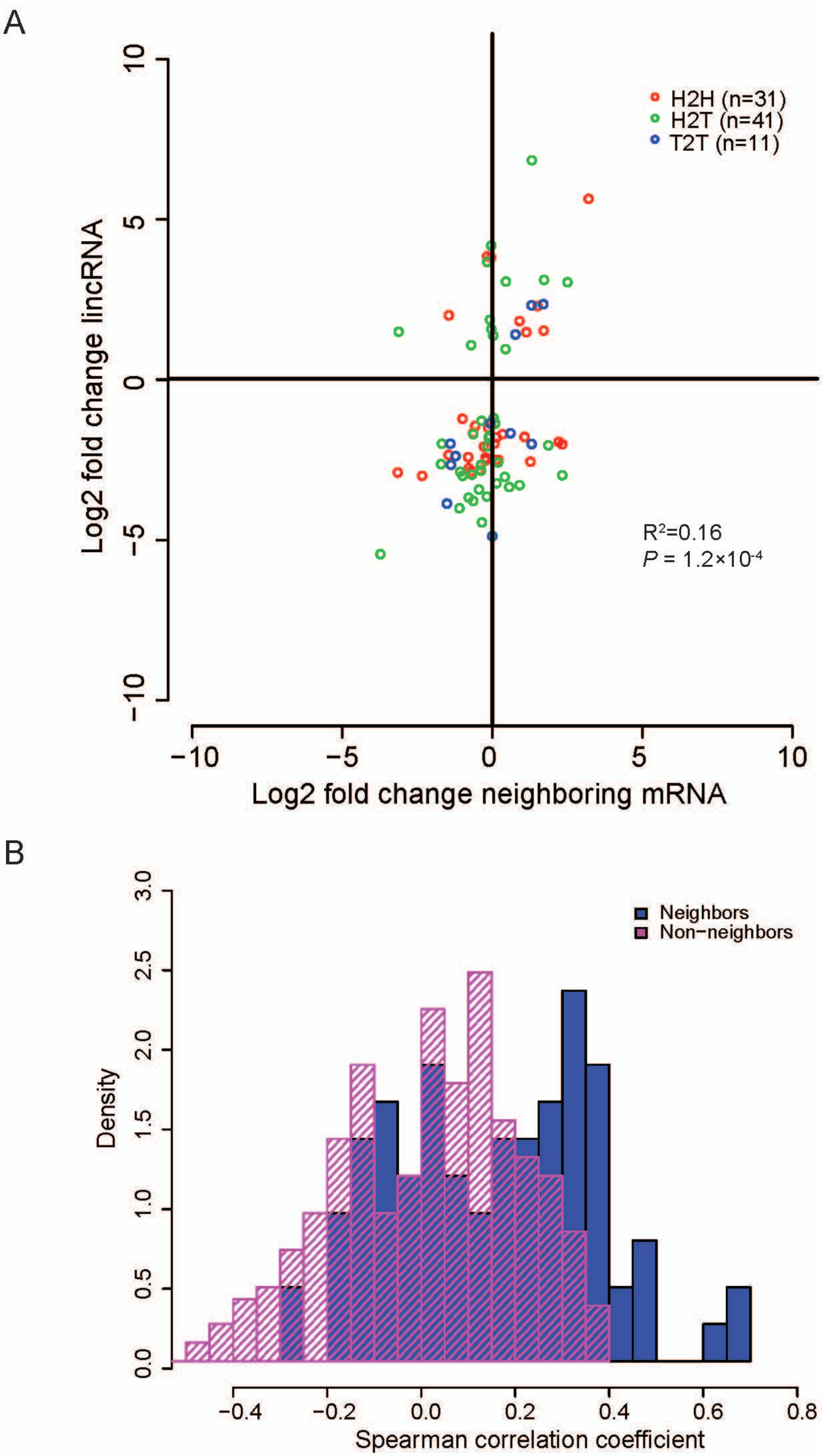

A
H2H (n=31)
H2T (n=41)
T2T (n=11)
R^2=0.16
P = 1.2×10^{-4}
Log2 fold change lincRNA
Log2 fold change neighboring mRNA
−10
−5
0
5
10
B
Neighbors
Non−neighbors
Density
Spearman correlation coefficient
0.0
0.5
1.0
1.5
2.0
2.5
3.0
−0.4
−0.2
0.0
0.2
0.4
0.6
0.8

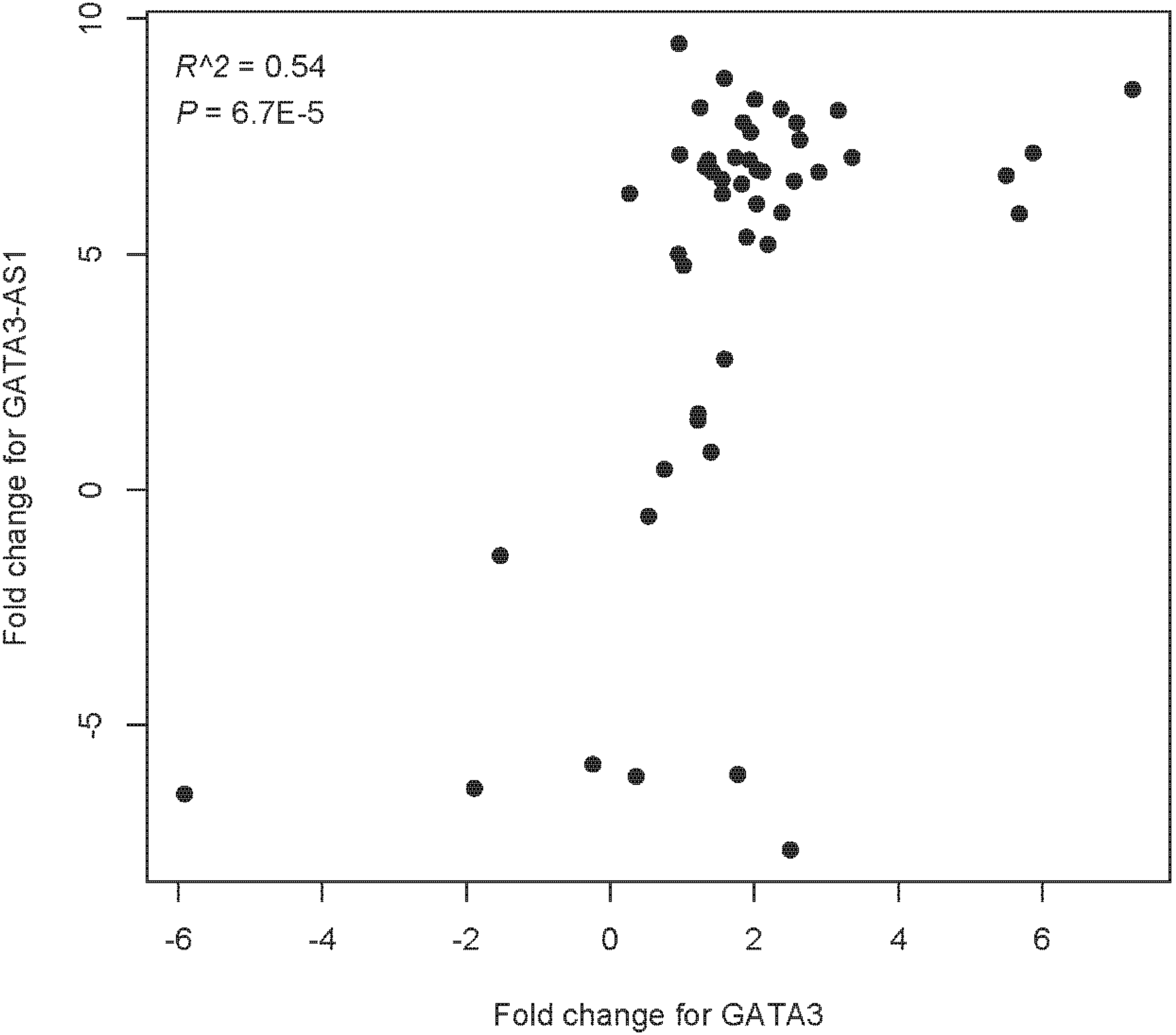
R^2 = 0.54
P = 6.7E-5
Fold change for GATA3-AS1
Fold change for GATA3
10
5
0
-5
-6
-4
-2
0
2
4
6

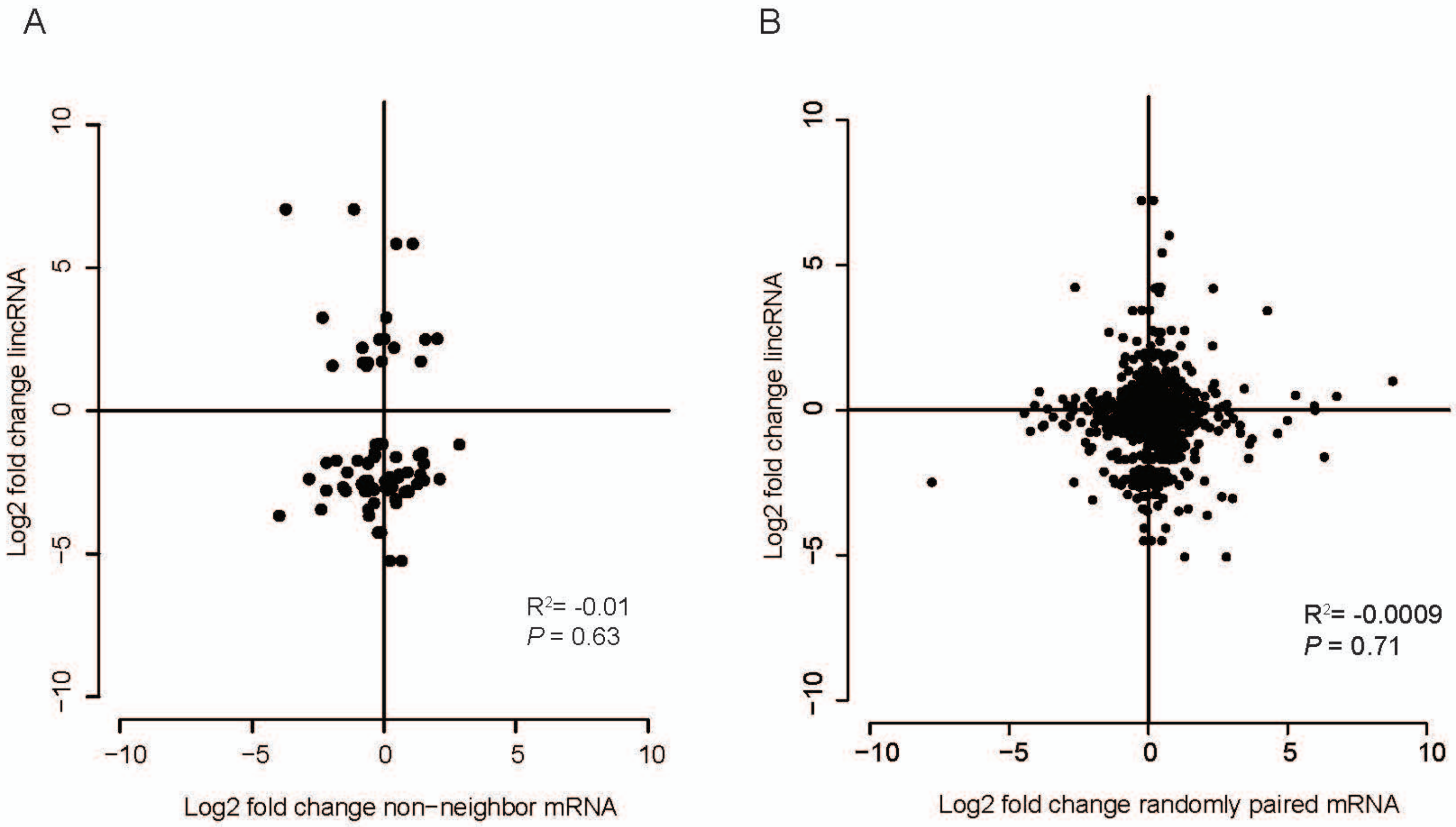

A
Log2 fold change lincRNA
Log2 fold change non−neighbor mRNA
R^2= -0.01
P = 0.63
B
Log2 fold change lincRNA
Log2 fold change randomly paired mRNA
R^2= -0.0009
P = 0.71

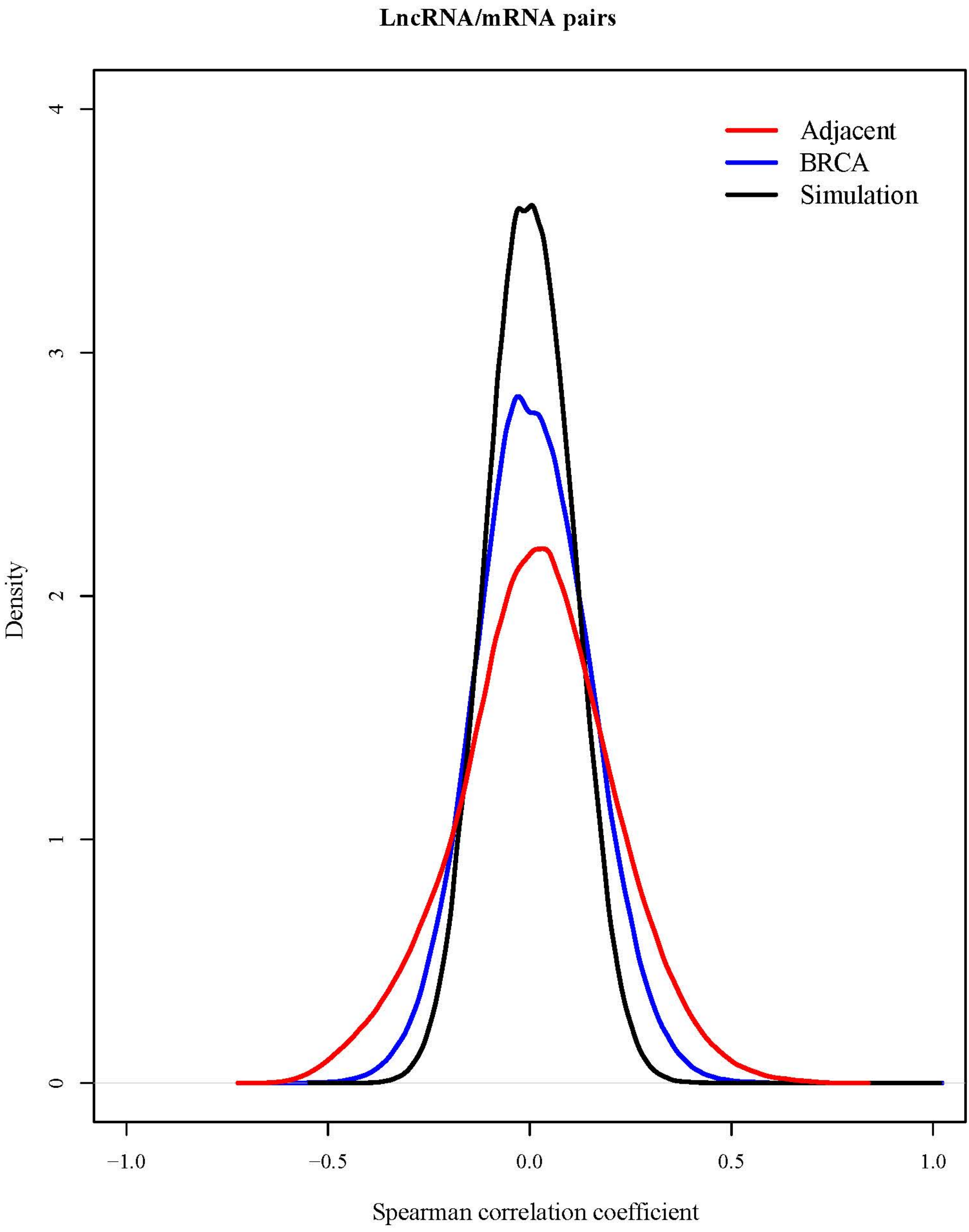
LncRNA/mRNA pairs
Adjacent
BRCA
Simulation
Density
0
1
2
3
4
−1.0
−0.5
0.0
0.5
1.0
Spearman correlation coefficient

Table S1: Summary of meta-data, including ChIP-seq data for transcription factor ERα and RNA-seq data used in this study

Sample ID	Description	Cell line	ER status	Series ID	Library strategy	Platform	PubMed ID
GSM1115990	ERa_ChIPSeq_repeat1	MCF-7	ER+	GSE45822	ChIP-seq	Illumina Genome Analyzer IIx	23728302
GSM1115991	ERa_ChIPSeq_repeat2	MCF-7	ER+	GSE45822	ChIP-seq	Illumina Genome Analyzer IIx	23728302
GSM1198711	ERa in MCF-7 Rep1	MCF-7	ER+	GSE49390	ChIP-seq	Illumina HiSeq 2000	24049078
GSM1198713	ERa in MCF-7 Rep2	MCF-7	ER+	GSE49390	ChIP-seq	Illumina HiSeq 2000	24049078
GSM665127	BT20_mRNA	BT20	ER-	GSE27003	RNA-Seq	Illumina Genome Analyzer II	21364760
GSM665128	BT474_mRNA	BT474	ER+	GSE27003	RNA-Seq	Illumina Genome Analyzer II	21364760
GSM665129	MCF10A_mRNA	MCF10A	ER-	GSE27003	RNA-Seq	Illumina Genome Analyzer II	21364760
GSM665130	MCF7_mRNA	MCF7	ER+	GSE27003	RNA-Seq	Illumina Genome Analyzer II	21364760
GSM665131	MDAMB231_mRNA	MDAMB231	ER-	GSE27003	RNA-Seq	Illumina Genome Analyzer II	21364760
GSM665132	MDAMB468_mRNA	MDAMB468	ER-	GSE27003	RNA-Seq	Illumina Genome Analyzer II	21364760
GSM665133	T47D_mRNA	T47D	ER+	GSE27003	RNA-Seq	Illumina Genome Analyzer II	21364760
GSM665134	ZR751_mRNA	ZR751	ER+	GSE27003	RNA-Seq	Illumina Genome Analyzer II	21364760
GSM984230	CAMA-1_mRNA-seq	CAMA-1	ER+	GSE28866	RNA-Seq	Illumina Genome Analyzer IIx	22929540
GSM984231	HCC1419_mRNA-seq	HCC1419	ER-	GSE28866	RNA-Seq	Illumina Genome Analyzer IIx	22929540
GSM984232	HCC1500_mRNA-seq	HCC1500	ER+	GSE28866	RNA-Seq	Illumina Genome Analyzer IIx	22929540
GSM984233	SUM_mRNA-seq	SUM	ER+	GSE28866	RNA-Seq	Illumina Genome Analyzer IIx	22929540
GSM984234	UACC-812_mRNA-seq	UACC-812	ER-	GSE28866	RNA-Seq	Illumina Genome Analyzer IIx	22929540
GSM984235	ZR-75-30_mRNA-seq	ZR-75-30	ER+	GSE28866	RNA-Seq	Illumina Genome Analyzer IIx	22929540

Table S2: LincRNAs validated in either tumor-adjacent normal paired samples or tumor-normal health women

LincRNA	Symbol	Tumor-Adjacent (n = 8		Tumor-Adjacent (*n* = 50)[b]		Tumor-Normal (*n* = 50)[c]	
		Log2-FC	*P*	Log2-FC	*P*	Log2-FC	*P*
ENSG00000261039	RP11-417E7	7.04	1.47E-08	7.11	6.45E-05	3.49	2.42E-03
ENSG00000230838	AC093850	5.84	6.87E-08	15.03	1.42E-11	9.72	1.19E-10
ENSG00000265185	SNORD3B-1	4.04	1.01E-06	4.59	4.97E-04	0.46	6.35E-01
ENSG00000262074	SNORD3B-2	3.87	2.43E-06	5.85	1.77E-04	-1.67	1.01E-01
ENSG00000224577	AC017048	3.26	4.31E-05	1.03	4.56E-01	2.17	2.69E-02
ENSG00000253364	RP11-731F5	2.52	4.88E-03	13.17	1.16E-08	9.31	1.78E-09
ENSG00000197308	GATA3-AS1	2.49	2.08E-05	5.58	1.43E-02	8.27	6.36E-07
ENSG00000259093	RP11-1112J20	2.20	6.87E-08	2.78	4.02E-06	2.02	8.65E-07
ENSG00000223573	TINCR	1.77	9.83E-06	1.45	1.76E-03	0.46	1.82E-01
ENSG00000230615	RP5-1198O20	1.72	2.80E-04	7.58	3.50E-09	6.53	1.75E-13
ENSG00000253161	RP11-150O12	1.68	1.00E-03	5.54	9.67E-04	4.61	4.12E-05
ENSG00000245750	RP11-279F6	1.57	1.17E-03	6.07	1.47E-03	4.96	1.63E-04
ENSG00000263500	CTB-104H12	1.27	1.28E-04	1.91	1.12E-01	-2.05	1.35E-02
ENSG00000261609	MIR4720	-1.09	2.20E-03	0.08	8.15E-01	-0.81	1.04E-02
ENSG00000214548	MEG3	-1.17	2.47E-07	-0.75	2.18E-01	-2.35	1.15E-07
ENSG00000237697	LINC00312	-1.18	9.13E-03	0.41	7.27E-01	-3.10	1.24E-04
ENSG00000267519	CTD-3252C9	-1.48	6.89E-11	1.33	2.00E-05	-1.01	4.12E-05
ENSG00000267272	RP5-1052I5	-1.56	2.64E-05	-2.37	3.67E-02	-3.02	2.65E-04
ENSG00000255455	RP11-890B15	-1.62	1.28E-04	-1.35	3.52E-02	-1.59	3.60E-04
ENSG00000234076	TPRG1-AS1	-1.74	3.52E-03	-3.37	2.06E-05	-1.93	1.12E-03
ENSG00000262728	AC123768	-1.82	4.90E-08	-3.01	2.80E-04	-1.58	1.27E-02
ENSG00000269930	RP11-932O9	-1.85	9.95E-07	-1.74	7.45E-03	-1.82	8.56E-05
ENSG00000260025	RP11-490M8	-2.15	1.10E-06	-2.78	6.05E-03	-1.99	1.18E-02
ENSG00000237357	RP11-475I24	-2.19	9.22E-05	-3.58	1.09E-04	-1.27	1.14E-01
ENSG00000260807	RP11-161M6	-2.24	1.64E-06	-5.65	1.34E-05	-1.96	4.49E-02
ENSG00000231246	RP5-965F6	-2.32	4.87E-07	-2.94	7.71E-02	-3.42	5.22E-03
ENSG00000266176	RP11-855A2	-2.39	9.44E-04	0.16	9.21E-01	-4.16	9.90E-05
ENSG00000260693	AC026150	-2.44	1.08E-06	-1.19	8.99E-02	-1.81	3.04E-04
ENSG00000223477	LINC00842	-2.46	8.86E-08	-5.50	1.07E-06	-2.93	7.21E-04
ENSG00000182021	RP11-381O7	-2.57	2.33E-05	-3.67	8.14E-05	-3.26	1.17E-06
ENSG00000228971	RP11-286B14	-2.64	4.20E-07	-4.25	7.17E-04	-1.76	7.07E-02
ENSG00000258498	DIO3OS	-2.68	1.01E-04	-3.76	9.62E-05	-3.34	2.49E-05
ENSG00000269936	MIRN145	-2.70	9.87E-13	-1.61	1.80E-03	-1.34	5.52E-04
ENSG00000229645	LINC00341	-2.77	6.89E-11	-2.82	1.50E-02	-3.89	7.12E-06
ENSG00000261064	RP11-1000B6	-2.79	9.95E-07	-1.77	6.81E-03	-1.33	7.23E-03
ENSG00000229108	AC005550	-2.81	2.28E-05	-6.34	7.85E-05	-4.31	4.11E-04
ENSG00000267194	RP1-193H18	-2.81	2.97E-06	-3.05	7.26E-04	-2.32	1.43E-03
ENSG00000260124	RP4-791K14	-2.84	8.18E-06	-2.32	4.72E-05	-1.84	8.56E-06
ENSG00000196972	LINC00087	-3.04	3.56E-06	-2.25	4.28E-03	-1.12	5.75E-02
ENSG00000224307	RP11-344B5	-3.10	7.24E-04	-1.88	3.44E-02	-2.07	2.87E-03
ENSG00000178947	LINC00086	-3.23	1.94E-09	-2.66	5.14E-03	-1.59	2.60E-02
ENSG00000259070	LINC00639	-3.45	2.88E-07	-3.48	1.40E-04	-2.30	8.87E-04
ENSG00000256124	RP11-84E24	-3.59	1.27E-05	-1.37	1.14E-02	-0.41	3.00E-01
ENSG00000271239	RP11-238F2	-3.67	3.37E-06	-3.86	5.24E-02	-3.19	3.08E-02
ENSG00000262179	RP1-302G2	-4.26	6.95E-07	-4.74	1.56E-03	-5.21	3.39E-06
ENSG00000232079	AL035610	-4.69	1.31E-06	-0.56	1.70E-01	-0.60	4.38E-02
ENSG00000246430	RP11-16M8	-5.25	2.61E-06	-3.19	3.97E-02	-6.36	4.27E-09

[a]Comparison from TCGA

[b,c]Comparison from IUBC

Table S3: Expression levels of 37 lincRNAs across 14 breast cancer cell lines

Symbol	CAMA-1	HCC1419	HCC1500	SUM	UACC-812	ZR-75-30	BT20	BT474	MCF10A	MCF-7	MDAMB231	MDAMB468	T47D	ZR751
RP11-417E7	0.01	0.01	0.01	0.01	0.01	0.01	0.01	0.01	1.051	0.01	0.01	0.01	0.01	0.01
AC093850	0.01	0.01	0.01	0.01	0.01	0.01	0.01	0.01	20.263	0.01	0.01	0.01	0.01	0.01
AC017048	0.01	0.01	0.01	0.01	0.01	0.01	0.01	0.01	0.01	0.01	0.01	0.01	0.01	0.01
RP11-731F5	0.01	0.01	0.01	0.01	0.01	0.01	0.01	0.01	0.01	0.01	0.01	0.01	0.01	0.01
GATA3-AS1	0.01	0.01	7.38	0.01	102.708	107.533	43.388	76.526	0.01	124.745	0.01	0.01	34.88	53.679
RP11-1112J20	0.01	0.01	0.01	0.01	0.01	0.01	0.01	0.01	0.01	0.01	0.01	0.01	0.01	0.01
RP5-1198O20	0.01	0.01	0.01	0.01	0.01	0.01	0.01	0.01	0.01	0.01	0.01	0.01	0.01	0.01
RP11-150O12	0.01	0.01	0.01	0.01	0.01	0.01	0.01	0.01	3.496	0.01	4.22	0.01	0.01	0.01
RP11-279F6	4.009	6.707	12.974	12.037	0.01	4.752	0.01	3.863	0.01	0.01	0.01	0.01	0.01	9.923
MEG3	0.01	0.01	0.01	0.01	0.01	0.01	0.01	0.01	0.01	0.01	0.01	0.01	0.01	0.01
LINC00312	0.01	0.01	0.01	0.01	3.247	1.988	0.01	0.01	1.152	0.98	0.01	0.01	1.3	0.01
CTD-3252C9	4.502	5.111	1.689	7.409	4.285	5.805	2.663	2.844	7.08	3.24	4.413	7.386	1.48	2.419
RP5-1052I5	0.01	0.01	0.01	0.01	0.01	0.01	0.01	0.01	0.01	0.01	0.01	0.01	0.01	0.01
RP11-890B15	2.198	3.076	3.242	0.01	4.961	0.01	1.03	1.192	1.89	0.841	2.48	5.782	2.629	2.462
TPRG1-AS1	0.01	0.01	0.01	0.01	0.01	0.01	0.01	0.01	0.01	0.01	0.01	0.01	3.623	0.01
AC123768	0.01	0.01	0.01	0.01	0.01	0.01	0.01	1.711	0.01	0.01	0.01	0.01	0.01	0.01
RP11-932O9	0.01	0.01	0.01	0.01	0.01	0.01	0.01	0.01	0.01	0.01	0.01	0.01	0.01	0.01
RP11-490M8	0.01	0.01	0.01	0.01	0.01	0.01	3.205	0.01	2.814	3.859	2.733	5.258	4.964	0.01
RP11-161M6	0.01	0.01	0.01	0.01	0.01	0.01	0.788	0.01	0.01	0.998	0.01	0.01	0.01	0.01
RP5-965F6	0.01	0.01	0.01	0.01	0.01	0.01	0.01	0.01	0.01	0.01	0.01	0.01	0.01	0.01
RP11-855A2	0.01	7.301	3.58	0.01	0.01	1.738	0.01	1.912	0.01	0.01	0.01	0.01	3.262	0.01
AC026150	0.01	0.01	0.01	0.01	0.01	0.01	0.01	0.01	0.01	0.01	0.01	0.01	0.01	0.01
LINC00842	0.01	0.01	0.01	0.01	0.01	0.01	5.901	0.01	5.195	2.232	2.147	6.005	0.01	0.01
RP11-381O7	0.01	0.01	0.01	0.01	0.01	0.01	0.01	0.01	0.01	0.01	0.01	0.01	0.01	0.01
DIO3OS	0.01	0.01	0.01	0.01	0.01	0.01	0.01	1.122	0.01	0.459	0.01	0.01	0.01	0.01
MIRN145	0.01	0.01	0.01	0.01	0.01	0.01	0.01	0.01	15.082	0.01	0.01	0.01	0.01	0.01
LINC00341	0.01	0.01	2.005	5.217	2.679	1.693	0.558	0.977	0.01	0.01	2.233	1.465	1.262	0.01
RP11-1000B6	0.01	0.01	0.01	0.01	0.01	0.01	0.01	0.01	0.01	0.01	0.01	0.01	2.545	0.01
AC005550	0.01	0.01	0.01	0.01	0.01	0.01	0.01	0.01	0.01	0.01	0.01	0.01	0.01	0.01
RP1-193H18	1.175	0.01	1.984	0.01	0.01	0.01	0.73	0.974	1.029	0.01	0.01	0.01	0.618	1.838
RP4-791K14	0.01	0.01	0.01	0.01	0.01	0.01	0.01	0.01	0.01	0.01	0.01	1.818	1.213	0.01
RP11-344B5	0.01	0.01	0.01	0.01	0.01	0.01	0.01	0.01	0.01	0.01	0.01	2.824	1.331	0.01
LINC00086	0.01	0.01	0.01	0.01	3.433	0.01	0.01	0.01	0.01	0.942	0.01	0.01	0.01	1.905
LINC00639	0.01	0.01	0.01	0.01	0.01	0.01	0.01	1.315	0.01	0.01	0.01	0.01	0.01	0.01
RP11-238F2	0.01	0.01	0.01	0.01	0.01	0.01	0.01	0.01	0.01	0.01	0.01	0.01	0.01	0.01
RP1-302G2	0.01	0.01	0.01	9.353	0.01	0.01	0.01	0.01	0.01	0.01	0.01	0.01	0.01	0.01
RP11-16M8	0.01	0.01	0.01	0.01	0.01	0.01	0.01	0.01	0.836	0.01	0.01	0.01	0.01	0.01

Table *S4:* Expression alteration of 26 transcription factors in 85 pairs of breast cancer and adjacent normal tissue

Gene Symbol	GeneID	BRCA	Adjacent Normal	Log2-FC	P.BH-adjst	Description
CEBPB	1051	20.426	23.668	-0.213	0.004	CCAAT/enhancer binding protein (C/EBP), beta
MYC	4609	36.375	75.807	-1.059	7.72E-09	v-myc avian myelocytomatosis viral oncogene homolog
CTCF	10664	17.096	15.972	0.098	0.121	CCCTC-binding factor
E2F1	1869	5.151	0.957	2.428	4.54E-14	E2F transcription factor 1
EGR1	1958	73.733	415.881	-2.496	6.40E-14	early growth response 1
ELF1	1997	21.914	21.877	0.002	0.717	E74-like factor 1
FOSL2	2355	19.468	21.654	-0.154	0.193	FOS-like antigen 2
FOXM1	2305	12.345	1.502	3.039	4.54E-14	forkhead box M1
GABPA	2551	7.179	9.108	-0.343	7.72E-09	GA binding protein transcription factor, alpha subunit 60kDa
GATA3	2625	149.624	50.414	1.569	5.24E-11	GATA binding protein 3
HDAC2	3066	14.921	10.509	0.506	1.54E-05	histone deacetylase 2
JUND	3727	47.142	83.805	-0.830	2.15E-10	jun D proto-oncogene
MAX	4149	28.833	25.098	0.200	0.004	MYC associated factor X
NR2F2	7026	19.872	14.555	0.449	0.040	nuclear receptor subfamily 2, group F, member 2
NRSF	5978	7.059	6.193	0.189	0.011	RE1-silencing transcription factor
EP300	2033	12.666	12.873	-0.023	0.404	E1A binding protein p300
PML	5371	9.408	9.252	0.024	0.733	promyelocytic leukemia
POLR2A	5430	34.076	31.752	0.102	0.359	polymerase (RNA) II (DNA directed) polypeptide A, 220kDa
RAD21	5885	108.087	46.992	1.202	1.91E-11	RAD21 homolog (S. pombe)
SIN3A	25942	13.650	12.610	0.114	0.359	SIN3 transcription regulator family member A
SRF	6722	11.162	16.958	-0.603	2.21E-09	serum response factor
TAF1	6872	6.307	7.330	-0.217	0.001	TAF1 RNA polymerase II, TATA box binding protein (TBP)-associated factor, 250kDa
TCF12	6938	15.633	16.318	-0.062	0.215	transcription factor 12
TCF7L2	6934	6.563	12.732	-0.956	1.35E-12	transcription factor 7-like 2 (T-cell specific, HMG-box)
TEAD4	7004	6.717	3.464	0.955	1.91E-11	TEA domain family member 4
ZNF217	7764	23.473	13.360	0.813	1.71E-08	zinc finger protein 217

Table *S5* : Functional prediction of 37 lincRNAs in both tumor and adjacent normal tissues

**Ensembl ID**	**Symbol**	**Functional enrichment in adjacent normal tissues**	**Functional enrichment in breast tumor**
ENSG00000261039	RP11-417E7	NA	cell adhesion
ENSG00000230838	AC093850	NA	cell adhesion
ENSG00000224577	AC017048	NA	potassium ion transport
ENSG00000253364	RP11-731F5	NA	immune response and positive regulation of immune system process
ENSG00000197308	GATA3-AS1	NA	defense response
ENSG00000259093	RP11-1112J20	cell cycle and division, proliferation	na
ENSG00000230615	RP5-1198O20	ectoderm development	na
ENSG00000253161	RP11-150O12	NA	na
ENSG00000245750	RP11-279F6	establishment of protein localization	cellular protein localization
ENSG00000214548	MEG3	NA	na
ENSG00000237697	LINC00312	NA	na
ENSG00000267519	CTD-3252C9	regulation of cell proliferation	anti-apoptosis
ENSG00000267272	RP5-1052I5	regulation of transcription	defense response
ENSG00000255455	RP11-890B15	NA	na
ENSG00000234076	TPRG1-AS1	positive regulation of apoptosis	na
ENSG00000262728	AC123768	NA	na
ENSG00000269930	RP11-932O9	translational elongation	na
ENSG00000260025	RP11-490M8	NA	na
ENSG00000260807	RP11-161M6	translational elongation and ribonucleoprotein complex biogenesis	na
ENSG00000231246	RP5-965F6	inflammatory and immune response	na
ENSG00000266176	RP11-855A2	RNA processing	na
ENSG00000260693	AC026150	regulation of Ras protein signal transduction	na
ENSG00000223477	LINC00842	excretion	na
ENSG00000182021	RP11-381O7	regulation of transcription	na
ENSG00000258498	DIO3OS	ribonucleoprotein complex biogenesis	na
ENSG00000269936	hsa-mir-145	RNA processing	na
ENSG00000229645	LINC00341	regulation of cell proliferation	na
ENSG00000261064	RP11-1000B6	induction of apoptosis	na
ENSG00000229108	AC005550	cell adhesion and Wnt signaling pathway	na
ENSG00000267194	RP1-193H18	posttranscriptional regulation of gene expression	na
ENSG00000260124	RP4-791K14	regulation of transcription	na
ENSG00000224307	RP11-344B5	NA	na
ENSG00000178947	LINC00086	translational elongation and ribonucleoprotein complex biogenesis	na
ENSG00000259070	LINC00639	NA	na
ENSG00000271239	RP11-238F2	translational elongation and ribonucleoprotein complex biogenesis	na
ENSG00000262179	RP1-302G2	regulation of transcription	na
ENSG00000246430	RP11-16M8	regulation of cell proliferation and cell migration	na

Table S6: Expression levels of 22 lincRNAs showing differential expression across breast cancer subtypes

**Ensembl ID**	**Symbol**	**Log2-FC**	**Chr**	**Start**	**End**	**LumiA**	**LumiB**	**Her2**	**Basal-like**
ENSG00000261039	RP11-417E7	7.04	chr6	169575400	169582835	0.81	-0.03	-0.29	-1.38
ENSG00000230838	AC093850	5.84	chr2	216582765	216584147	0.14	0.28	0.28	-1.03
ENSG00000224577	AC017048	3.26	chr2	177502437	177520686	1.17	0.19	-1.69	-3.12
ENSG00000253364	RP11-731F5	2.52	chr14	106110832	106115394	-1.58	-1.02	2.16	0.88
ENSG00000197308	GATA3-AS1	2.49	chr10	8092412	8095447	0.61	0.95	-2.77	-4.21
ENSG00000259093	RP11-1112J20	2.2	chr14	63589750	63594932	-0.1	-0.2	0.19	0.32
ENSG00000253161	RP11-150O12	1.68	chr8	37278858	37411701	-0.45	1.08	-0.27	-1.50
ENSG00000245750	RP11-279F6	1.57	chr15	69755364	69863775	0.68	0.39	0.18	-5.71
ENSG00000214548	MEG3	-1.17	chr14	101245746	101327368	0.56	-0.1	0.24	-0.98
ENSG00000237697	LINC00312	-1.18	chr3	8613467	8615561	0.1	0.35	-0.29	-0.65
ENSG00000267519	CTD-3252C9	-1.48	chr19	13945329	13947103	0.11	-0.45	-0.24	0.60
ENSG00000255455	RP11-890B15	-1.62	chr11	130736148	130740142	0.38	0.23	-0.29	-0.95
ENSG00000234076	TPRG1-AS1	-1.74	chr3	188659503	188665428	0.32	0.04	-0.23	-0.44
ENSG00000260025	RP11-490M8	-2.15	chr2	36581891	36582257	0.04	-0.81	-0.29	1.08
ENSG00000260807	RP11-161M6	-2.24	chr16	1025760	1031596	-2.35	-2.69	-2.21	4.11
ENSG00000266176	RP11-855A2	-2.39	chr17	65989995	65992547	-0.63	1.65	-0.99	-2.57
ENSG00000229645	LINC00341	-2.77	chr14	95873605	95876427	0.33	-0.43	-0.07	0.25
ENSG00000267194	RP1-193H18	-2.81	chr17	67547498	67550002	0.04	-0.47	0	0.62
ENSG00000260124	RP4-791K14	-2.84	chr20	47980422	47984296	-1.27	-0.71	-0.71	2.00
ENSG00000224307	RP11-344B5	-3.1	chr9	132044736	132048007	0.64	-0.36	-0.68	-0.05
ENSG00000178947	LINC00086	-3.23	chrX	134555867	134559682	0.1	-0.71	0.1	0.79
ENSG00000262179	RP1-302G2	-4.26	chr6	44184675	44185901	0.18	0.35	0.26	-1.32
